# Visualizing flow in an intact CSF network using optical coherence tomography: implications for human congenital hydrocephalus

**DOI:** 10.1038/s41598-019-42549-4

**Published:** 2019-04-17

**Authors:** Priya Date, Pascal Ackermann, Charuta Furey, Ina Berenice Fink, Stephan Jonas, Mustafa K. Khokha, Kristopher T. Kahle, Engin Deniz

**Affiliations:** 1 Pediatric Genomics Discovery Program, Yale University School of Medicine, 333 Cedar Street, New Haven, CT 06510 USA; 2 Department of Pediatrics, Yale University School of Medicine, 333 Cedar Street, New Haven, CT 06510 USA; 3 Department of Medical Informatics, Uniklinik RWTH Aachen, Pauwelsstr 30, 52074 Aachen, Germany; 4 Department of Genetics, Yale University School of Medicine, 333 Cedar Street, New Haven, CT 06510 USA; 5 Department of Informatics, Technical University of Munich, Munich, Germany; 6 Department of Neurosurgery and Cellular & Molecular Physiology, and Centers for Mendelian Genomics, 333 Cedar Street, New Haven, CT 06510 USA

**Keywords:** High-throughput screening, Disease model

## Abstract

Cerebrospinal fluid (CSF) flow in the brain ventricles is critical for brain development. Altered CSF flow dynamics have been implicated in congenital hydrocephalus (CH) characterized by the potentially lethal expansion of cerebral ventricles if not treated. CH is the most common neurosurgical indication in children effecting 1 per 1000 infants. Current treatment modalities are limited to antiquated brain surgery techniques, mostly because of our poor understanding of the CH pathophysiology. We lack model systems where the interplay between ependymal cilia, embryonic CSF flow dynamics and brain development can be analyzed in depth. This is in part due to the poor accessibility of the vertebrate ventricular system to *in vivo* investigation. Here, we show that the genetically tractable frog *Xenopus tropicalis*, paired with optical coherence tomography imaging, provides new insights into CSF flow dynamics and role of ciliary dysfunction in hydrocephalus pathogenesis. We can visualize CSF flow within the multi-chambered ventricular system and detect multiple distinct polarized CSF flow fields. Using CRISPR/Cas9 gene editing, we modeled human L1CAM and CRB2 mediated aqueductal stenosis. We propose that our high-throughput platform can prove invaluable for testing candidate human CH genes to understand CH pathophysiology.

## Introduction

Cerebrospinal fluid (CSF) provides protection for the brain in the rigid cranium, clears waste produced by brain cells, and circulates growth factors important for neurodevelopment^1^. After neural tube closure, CSF is secreted primarily by the choroid plexus in the lateral ventricles, flows sequentially through the third and fourth ventricles, and then is reabsorbed by the arachnoid granulations and the lymphatic system^2^. The cilia on ependymal cells that line the ventricular cavities are important for CSF production and CSF flow through the ventricles^3–6^. As the choroid plexus pulsation and the wall motions dominates CSF dynamics in the center regions of the ventricles, ependymal cilia dominates near-wall cerebrospinal fluid dynamics in the lateral ventricles^7^ and regulates neuroblast migration^8^. Based on human and mouse genetics, mutations in genes that alter the structure and/or function of cilia can cause congenital hydrocephalus (CH); however, the mechanism of these genes on disease pathogenesis, including physiological embryonic CSF flow patterns, are poorly understood^9–14^.

CSF flow is critical for proper neurogenesis as it regulates neuroblast migration^8^. Understanding interactions between CSF flow and brain development is important to decipher the pathogenesis of hydrocephalus. While recent studies in single brain ventricular explants have highlighted potential complexities of CSF flow fields^15^, a global understanding of these networks in an intact, multi-compartmental ventricular system remains rudimentary. This is in part due to technical limitations of accessing the brain ventricles in a live animal through the rigid cranial vault and multilayered cortex, which impedes real-time imaging. Importantly, studies in other organ systems with cilia-lined fluid cavities (e.g., the fallopian tube or upper respiratory tract) have revealed the importance of the interaction between the cilia, chamber geometry, and fluid viscosity on the mechanical polarization of the fluid-flow. This is a severe limitation for explant experiments that are widely used to understand the polarized CSF circulation^16–19^. Therefore, studying explants of only one chamber of a post-mortem ventricle is suboptimal for analyzing the CSF flow network. Unfortunately, the lack of model systems enabling real time changes in CSF flow pattern and ventricular development has been a critical roadblock in understanding the pathogenesis of hydrocephalus.

The frog *Xenopus tropicalis* has a particular advantage for studying brain ventricular development and associated CSF flow dynamics. At stage 45 (Nieuwkoop - Faber staging. ~ post fertilization day 4 at 25 °C), the *Xenopus* brain is transparent, enabling Optical Coherence Tomography (OCT), a cross-sectional imaging modality that dynamically detects *in vivo* morphology at micrometer resolution^20–23^. We recently demonstrated that OCT imaging effectively detects structural changes in *Xenopus* craniofacial and cardiac structures, a useful screening tool for human genetic cranio-cardiac malformation research^24^. Here, we expand this platform to analyze *in vivo* CSF dynamics during ventricular development and use this approach to better understand the pathogenesis of patients with congenital hydrocephalus.

We show that *Xenopus*, coupled with OCT imaging, enables the simultaneous visualization of brain ventricle development, CSF flow dynamics, and ependymal cilia function. Fortuitously, we take advantage of endogenous, free-floating intraventricular particles for particle tracking revealing multiple, differentially polarized flow fields throughout the ventricular system. By adding F0 CRISPR, we can rapidly evaluate the impact of gene depletion on CSF flow and ventricular structure. By targeting human congenital hydrocephaly candidate genes, we show that we can recapitulate the aqueductal stenosis in frogs, as seen in non-communicating type of human hydrocephalus. Lastly, we gain insight into the pathogenesis of hydrocephalus by modeling the effects of the candidate CH disease genes. This work establishes a platform for the rapid testing of candidate human CH genes, expeditious given the recent large-scale sequencing efforts of this disease^25^.

## Results

### *Xenopus* CNS structural and CSF flow imaging

**Figure 1:**
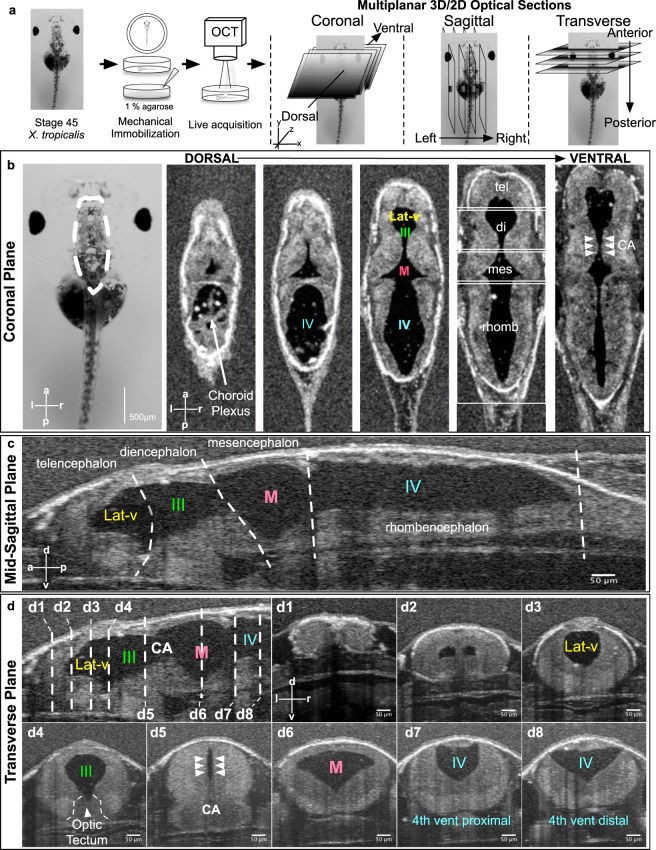
*In Vivo* OCT imaging of the *Xenopus* tadpole ventricular system. (**a**) Stage 45 tadpoles are embedded in 1% low-melt agarose with the dorsal side of the animal facing the OCT beam. 2D and 3D images are taken. (**b**) A stage 45 tadpole as seen under light microscopy. Far left panel - white dotted line outlines the brain. A series of OCT images taken at progressively more ventral planes show the ventricular system with different parts of the brain. (**c**) A mid-sagittal view of the ventricular system at the midline. White dotted lines show the boundaries between different brain regions. (**d**) Transverse sections through the ventricular system along the anterior to posterior axis of the animal (d1–d8). The position of each section corresponds to the white dotted lines shown on the mid-sagittal view in the top left panel. (Lat-V: lateral ventricle, III: 3^rd^ ventricle, M: Midbrain ventricle, IV: 4^th^ ventricle, tel: Telencephalon, di: Diencephalon, mes: Mesencephalon, rhomb: Rhombencephalon, CA: Cerebral aqueduct).

We applied OCT imaging to *Xenopus* tadpoles *in vivo* by embedding stage 45 tadpoles (post fertilization day 4 raised at 25 °C) in an agarose gel (1%) with the dorsal CNS structures positioned towards the OCT beam (Fig. 1a). This is a non-destructive, *in vivo* method of imaging as the tadpoles can be easily recovered from the agarose gel and raised to adulthood. Optical coherence imaging utilizes the information from backscattered light to create an image of the underlying tissue. It is analogous to clinical ultrasound but uses light. A single OCT depth profile is called an A-scan. When this A-scan is swept back and forth, the OCT system can create a 2D image (a B-scan). By combining multiple 2D images, the OCT system can also generate a 3D image of the underlying structure.

For our purposes, the embryo is oriented such that the left-right axis is aligned along the x direction, the anteroposterior axis is aligned along the y direction, and the dorsal-ventral axis is aligned in the z direction (Fig. 1a). We then divided our analysis into 3D structural and 2D CSF flow imaging.

#### 3D Structural Imaging

Once the CNS structures are captured by OCT imaging, we generate a 3D dataset that can be examined in three orthogonal, cross-sectional planes (Fig. 1b–d – Movies 1–2). In addition, we can isolate and measure specific ventricle volumes (Fig. S1d). In different planes, different anatomical structures can be readily isolated and measured:

1. Coronal plane (Fig. 1b) – Using this plane, we can visualize the four ventricles (lateral, 3^rd^, midbrain and 4^th^ ventricle) in the xy-plane as we make 2D sections through the z-axis. To measure ventricular diameter, the user can examine each optical slice through the Z axis for the largest diameter for quantification (Figs 1b – S1b). Furthermore, the ventricular connections (e.g. cerebral aqueduct [CA]) can be visualized along the z-axis. Here, we should also note that as development progress in frogs, two lateral ventricles form (Fig. 1–d2) budding from the 3^rd^ ventricle. At the tadpole stages we are imaging, they are visible as one and referred as one ventricle throughout the paper.
2. Mid-sagittal plane (Fig. 1c) – In this plane, we can visualize all four ventricles in a dorsal-ventral (DV) view along the anterior-posterior (AP) axis. We can outline the telencephalon, diencephalon, mesencephalon and rhombencephalon along with the associated ventricles. This plane is useful in examining CSF flow dynamics in each ventricle and also in relation to the adjacent ventricular system via the aqueducts. In this plane, we can also measure the total ventricular length along the AP axis (Figs 1c – S1a).
3. Transverse plane (Fig. 1d) – In this plane, we can measure the maximal dorsal-ventral (DV) length and the maximal width along the x-axis for each ventricle (Figs 1d- S1c). The transverse plane is particularly useful to measure the width and the DV length of the cerebral aqueduct that will eventually form the *Xenopus* equivalent of the aqueduct.

#### 2D Imaging and particle tracking

**Figure 2:**
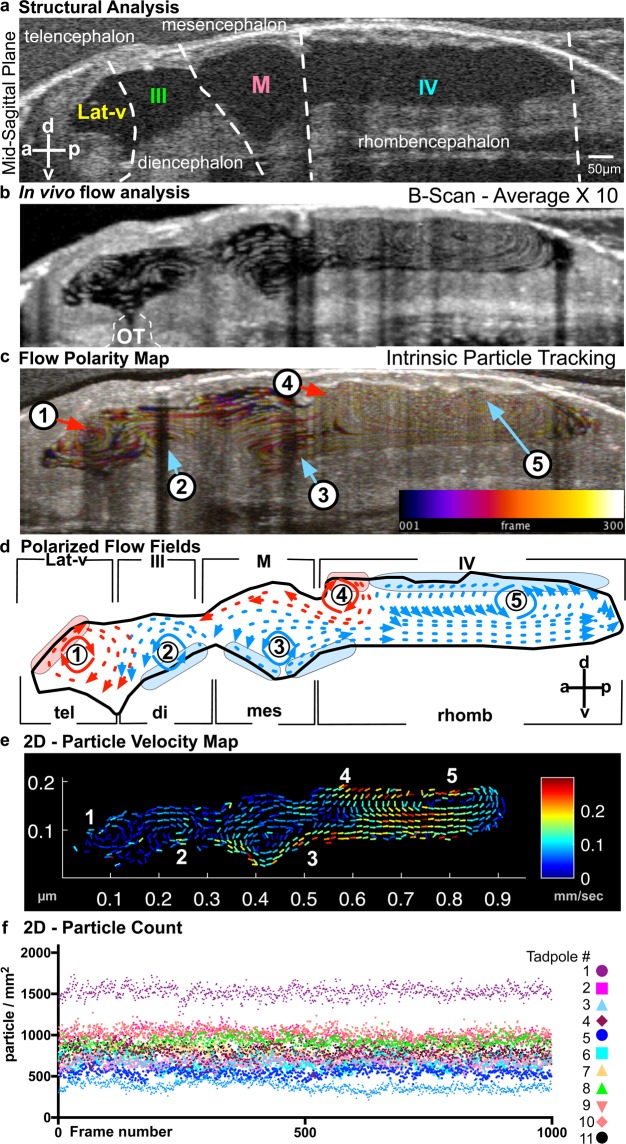
Imaging ventricular CSF flow with OCT. (**a**) Mid-sagittal view of the brain allowing simultaneous visualization of the four ventricles. (**b**) B-scan image acquisition using OCT: Ventricles of a stage 45 tadpole harbor native free floating particles. In this image, the spatial position of the particles are recorded over time to demonstrate discrete flow patterns within each ventricle (OT: optical tectum). (**c**) Intrinsic Particle Tracking: Using ImageJ, we tracked particles using 300 frames of 2D images. Temporal color coding depicts their trajectory over time. Color bar represents color versus corresponding frame number in the color-coded image. Based on the trajectory map, 5 distinct flow fields were observed: FF1-FF5. We numbered flow fields 1 to 5 along the anterior-posterior axis. FF 1-Telencephalic flow and FF 2-Diencephalic flow are in the lateral ventricle. FF 3-Mesencephalic flow is in the 3^rd^ ventricle. Finally, the 4^th^ ventricle has 2 flow fields: FF 4-Anterior Rhombencephalic flow and FF 5-Posterior Rhombencephalic flow. (**d**) Outline of the ventricular system and vector maps of the CSF flow fields. Based on the trajectory map and real time observation (Movie 3); FF1 and FF4 are clockwise and FF2, 3 and 5 are counter-clockwise. (**e**) 2D Particle Velocity Map: Particle trajectories averaged across all frames (1000) to form flow velocities (see methods for details). Based on the particle velocity map and real time observations FF1 and FF2 are relatively slower compared to FF 3, 4 and 5. The fastest flow is recorded within the 4^th^ ventricle. (**f**) 2D Particle Count (n = 11): Over 1000 frames, total particle number are counted for 11 different wild type tadpoles and plotted for each frame.

With 2D-cross sectional imaging, the scanning is fast enough to capture CSF flow. In the mid-sagittal plane where all four ventricles can be visualized simultaneously, we generated a global flow map. We took advantage of endogenous free-floating particles within the fluid-filled cavities of the ventricles. These particles appear in the range of 3.57–3.88 microns in diameter and can be readily resolved by OCT imaging. Using particle tracking techniques, we generated a CSF flow map (Fig. 2; see Methods for details) in the mid-sagittal plane. We mapped particle tracks over time in two ways: (1) B-Scan Average – We averaged 10 serial B-Scan images to form a map of the particle shift as a function of time (Fig. 2b). (2) Temporal color coding - We also used temporal color-coding to track particles over time. As a particle changes position it is coded with a different (warmer) color (ImageJ - see methods) (Fig. 2c). Based on these images, we discovered five discrete CSF “flow fields” (FFs) (Fig. 2d).

We expected CSF to flow along a major, single axis from the forebrain to the spinal cord; instead, we observed multiple flow fields that are clearly polarized independently with distinct velocities within different parts of the developing ventricle (Fig. 2d,e). While CSF flow fields were recently described in mammalian third ventricles^15^, differential polarization between the ventricles was unexpected and requires precise organization of the ependymal cells that drive flow. In describing the different flow fields, we oriented the embryo such that anterior is to the left and posterior is to the right with dorsal to the top in all of the following sections. In analyzing these images, we also quantified the endogenous particle counts for each frame and didn’t detect any significant variation between frames (1000 frames/acquisition/15 sec) or different tadpoles at the same stage (Fig. 2f).

FF1 - Telencephalic flow: This most anterior flow field is mostly confined to the telencephalon. On the mid-sagittal cross section, vector mapping shows a circular clockwise flow field along the anterior-posterior axis. The flow propels CSF anteriorly to the future lateral horns, inferiorly to the preoptic region and posteriorly towards the 3^rd^ ventricle. Based on the velocity maps, FF1 is significantly slower than the mesencephalic and rhombencephalic flows (Fig. 2b–e – Movie 3).

FF2 - Diencephalic flow: This flow field forms superior to the zona limitans intrathalamica, a cell lineage restriction boundary between the vertebrate thalamus and the prethalamus, which is important for diencephalon development. Unlike FF1, the diencephalic flow field is counter-clockwise so that the anterior part of FF2 reinforces with the posterior part of FF1 to direct CSF towards the preoptic region. At the superior aspect, most of the flow is directed anteriorly from the cerebral aqueduct to the lateral ventricle. Together, FF1 and FF2 unite in the midst of the lateral ventricle and direct CSF flow ventrally (Fig. 2b–e – Movie 3). Similar to FF1, flow velocities are relatively low.

FF3 - Mesencephalic flow: The third flow field forms at the ventral surface of the midbrain ventricle just superior to the ventral tegmentum. This circular flow field is polarized counterclockwise. Superiorly CSF flow is directed to the lateral and third ventricle via the cerebral aqueduct and inferiorly to the fourth ventricle (Fig. 2b–e – Movie 3). Based on the flow velocities, dorsal flow (aimed towards the lateral ventricle) is significantly slower than the ventral flow (aimed towards the 4^th^ ventricle). We recorded the slowest flow within the dorsal aspect of the midbrain ventricle.

FF4 - Anterior rhombencephalic flow: Interestingly, two discrete flow fields are observed within the rhombencephalon, each with an opposite polarization. Immediately posterior to the cerebellar region, the dorsal ventricular surface generates a clockwise flow field in which CSF is directed anteriorly to the midbrain ventricle (Fig. 2b–e – Movie 3).

FF5 - Posterior rhombencephalic flow: The posterior portion of the rhombencephalon contains the largest and fastest flow field, which flows counterclockwise. At the ventral aspect, CSF is directed posteriorly towards the spinal cord that is augmented with CSF flow originating at the posterior aspect of FF3 (Fig. 2b–e – Movie 3).

Taken together, these results demonstrate that OCT imaging can reliably detect CNS structures including the cerebral ventricles and CSF flow patterns in *Xenopus* tadpoles. Embryonic CSF flow in *Xenopus* is not uniform; there are multiple flow fields that allow continuous mixing of CSF between the ventricles. Since multiple flow fields are differentially polarized even within the same ventricle, there must exist a corresponding functional organization of ependymal cilia to generate this flow. Even at close proximity, these cells must drive extracellular fluid flow in opposite directions, suggesting a tight spatial control of the organization of ependymal ciliated cells.

### Loss of cilia motility or biogenesis causes discrete ventricular phenotypes

**Figure 3:**
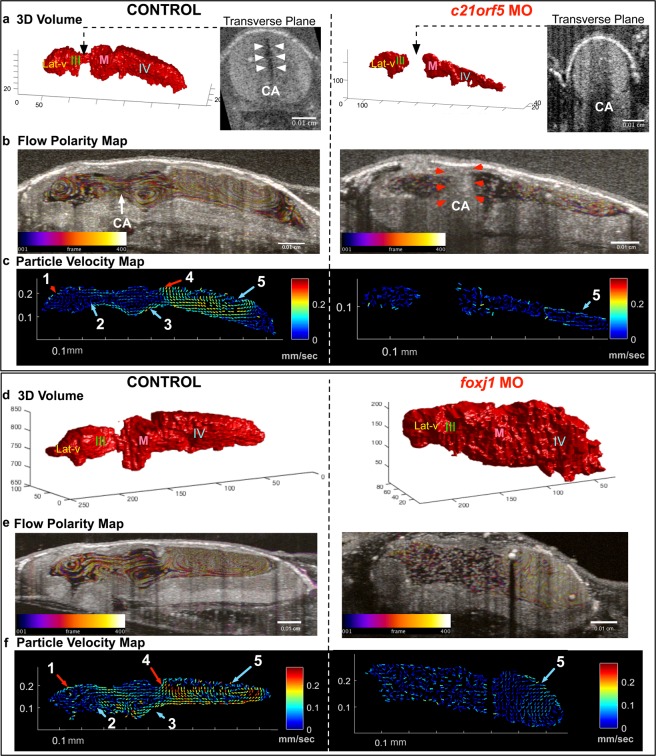
Characterization of *c21orf59* and *foxj1* morphants. (**a**) 3D rendering of the tadpole ventricular system shows aqueductal stenosis and a smaller ventricular system in *c21orf59* morphants compared to controls and (**b**,**c**) flow polarity and particle velocity maps confirm loss of FFs 1-4 as well as diminished flow in FF5. (**d**) 3D rendering of the tadpole ventricular system shows ventriculomegaly in *foxj1* morphants and (**e**,**f**) flow polarity and particle velocity map confirm loss of FFs1-4 and slow FF5. (Lat-V: lateral ventricle, III: 3^rd^ ventricle, M: Midbrain ventricle, IV: 4^th^ ventricle).

To test the sensitivity of OCT based particle tracking in low or no flow states and also to analyze the effects of the ependymal ciliary dysfunction on CSF flow dynamics and ventricle morphology, we perturbed cilia motility by knocking down *c21orf59*, which regulates the assembly of the outer dynein arms that drive ciliary beating^26^. These tadpoles demonstrated no or very slow CSF flow velocities as detected by OCT imaging (Fig. 3b,c). The ventricles were smaller in size and showed aqueductal stenosis between the lateral & 3^rd^ ventricles and midbrain ventricle (Figs 3a,b, S2a, S3a), which can lead to a form of non-communicating CH. This data is in agreement with mouse experiments where, loss of cilia motility genes Ccdc39 and Mdnah5 also result in hydrocephaly^3,5^. In comparison, to explore changes with loss of ependymal cilia, we knocked down *foxj1*, a master regulator of motile cilia biogenesis^27–29^. We observed a dramatic loss of flow throughout the ventricles, but unlike the *c21orf59* knockdown, the ventricles were significantly larger, resembling communicating hydrocephalus (Figs 3d,e, S2b,S3a). Of note, in both cases, despite the dramatic loss of cilia driven flow, FF5 remained partially intact (Figs 3c,f, S2a,b).

Ependymal cilia drive CSF flow but this is not the only mechanism. Another contributor to CSF flow is the choroid plexus; a network of blood vessels and modified ependymal cells that produce CSF. In tadpoles, FF5 is adjacent to where the choroid plexus forms. As these cells are also ciliated, it is difficult to differentiate to what degree FF5 is a function of the ciliated surface or CSF secretion by the plexus. Clearly in both morphants, FF5 velocities were diminished but not lost even in cases where the cilia were disrupted (*foxj1*MO). Based on these findings, we speculate that CSF secretion via the choroid plexus may be contributing to FF5 formation. Future work can explore the contribution of the choroid plexus to CSF circulation at these early stages of development.

### Loss of *L1CAM* function in *Xenopus* tadpoles causes aqueductal stenosis

Having established that we can readily visualize CSF flow, aqueductal stenosis, and communicating hydrocephalus, we next wanted to test if we model CH by depleting an established CH disease gene. In humans, loss-of-function (LOF) mutations in *L1CAM* cause X-linked hydrocephalus associated with stenosis of the cerebral aqueduct (OMIM#307000) and is the most common heritable form of hydrocephalus^30^. Several *l1*cam mouse knockouts exist with varying degrees of hydrocephalus, with or without aqueductal stenosis^31^. L1CAM is an integral membrane glycoprotein that mediates cell-to-cell adhesion at the cell surface but is not involved directly in cilia structure or function^32^. We depleted *Xenopus l1cam* using F0 CRISPR-Cas9. Briefly, we injected a guide RNA(sgRNA) and Cas9 protein into fertilized 1 cell embryos and raised F0 CRISPR mutant tadpoles to stage 45 (Day 4 at 25 °C) for OCT imaging. Injection of a sgRNA and Cas9 protein can readily lead to biallelic frameshift mutations in just a few hours after injection (Fig. S4a)^28^.

**Figure 4:**
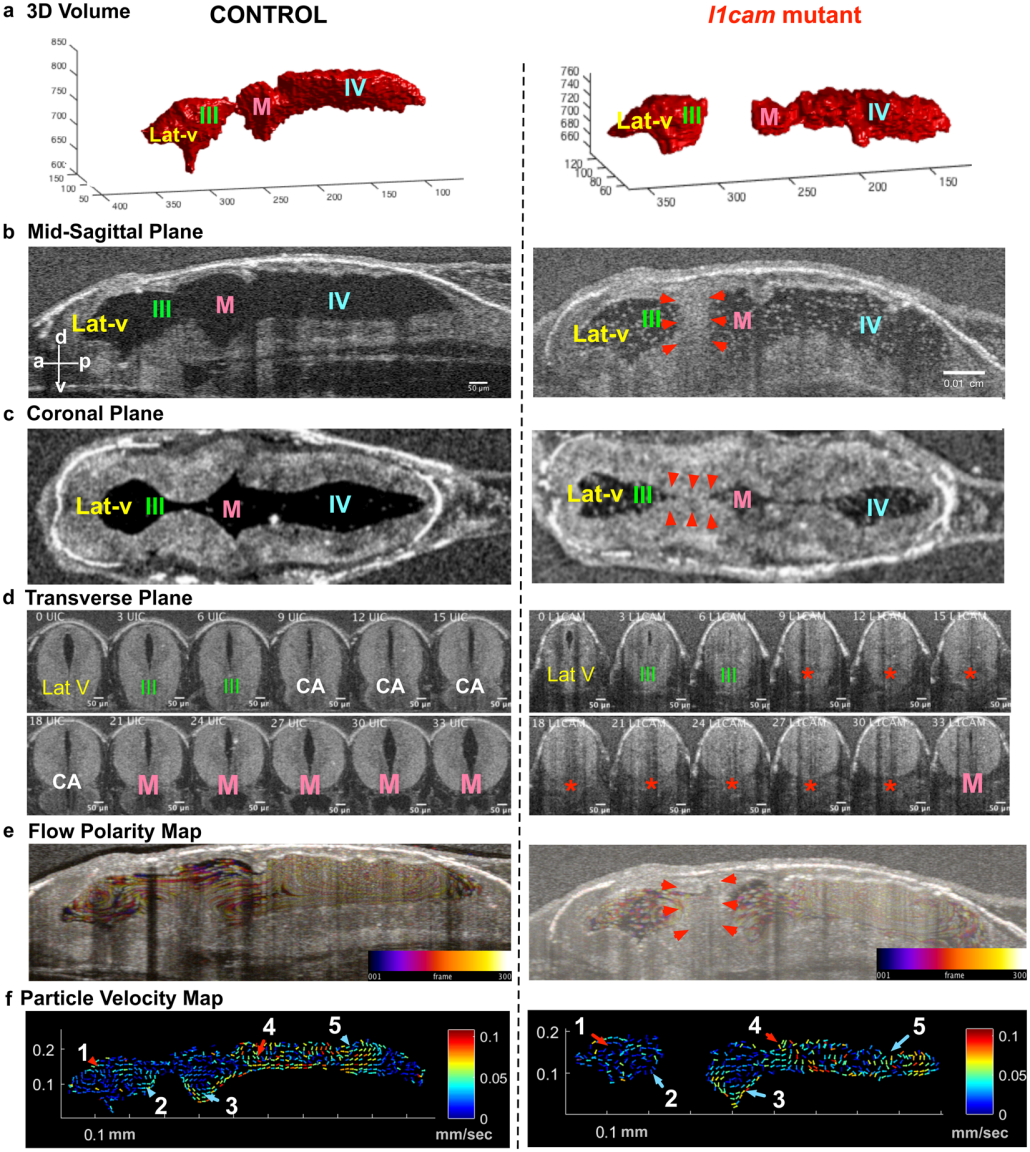
F0 CRISPR mutation in L1CAM causes cerebral aqueduct stenosis. The left column shows control and right column shows *l1cam* F0 CRISPR mutant images for all panels. (**a**) 3D rendering of the tadpole ventricular system shows aqueductal stenosis and a smaller ventricular system in *l1cam* F0 CRISPR mutant. (**b**) Mid sagittal view and (**c**) Coronal view of control and *l1cam* F0 CRISPR mutant, the later showing stenosis of the cerebral aqueduct (red arrowheads). (**d**) Transverse view of the control and *l1cam* F0 CRISPR mutant, starting at the end of the lateral ventricle through the cerebral aqueduct and ending in the midbrain ventricle. Control embryo shows normal opening of the duct whereas the mutant shows complete blockage (red star). (**e**) Relatively normal ciliary flow fields in the control and mutant animals. (**f**) 2D Particle Velocity Map shows intact FFs 1-5. (Lat-V: lateral ventricle, III: 3^rd^ ventricle, M: Midbrain ventricle, IV: 4^th^ ventricle).

Most of the *l1cam* F0 CRISPR mutant tadpoles had normal external morphology with some narrowing of the cranium. To test for gene modification and depletion of gene product, we used a T7 Endonuclease I assay and detected modified alleles at the *l1cam* locus with F0 CRISPR and also saw a loss of anti-L1cam antibody signal in F0 CRISPR embryos (Fig. S4a,c). With OCT imaging, we detected cerebral aqueductal stenosis in *l1cam* F0 CRISPR mutant tadpoles (Fig. 4a–d) using both of the non-overlapping sgRNAs that we tested (Fig. S5). Overall, the length of each ventricle in its anterior to posterior aspect in the mid-sagittal plane was slightly shorter and smaller in the F0 CRISPR mutants as compared to the controls (Fig. S3a,b–d). In ~50% of the *l1cam* F0 CRISPR mutants, we detected complete stenosis of the cerebral aqueduct, while the remaining 50% showed a narrowing (Figs 4d, S3a,b–d). Next, we investigated the ventricular CSF flow and mapped FF1 to FF5, which appeared intact (Figs 4e,f, S2c, Movie 5).

From these results, we conclude that depletion of *l1cam* in *Xenopus* obstructs the communication between the lateral - 3^rd^ and midbrain- 4^th^ ventricles due to cerebral aqueduct stenosis without a major impact on the FF in the ventricles. Thus, *Xenopus* can also be used to model non-communicating hydrocephalus when formed independent of ciliary dysfunction and it remains to be seen if such phenotypes affects ependymal cilia flow in later development.

### *CRB2* loss-of-function leads to cerebral aqueduct stenosis and ventriculomegaly in humans and frogs

**Figure 5:**
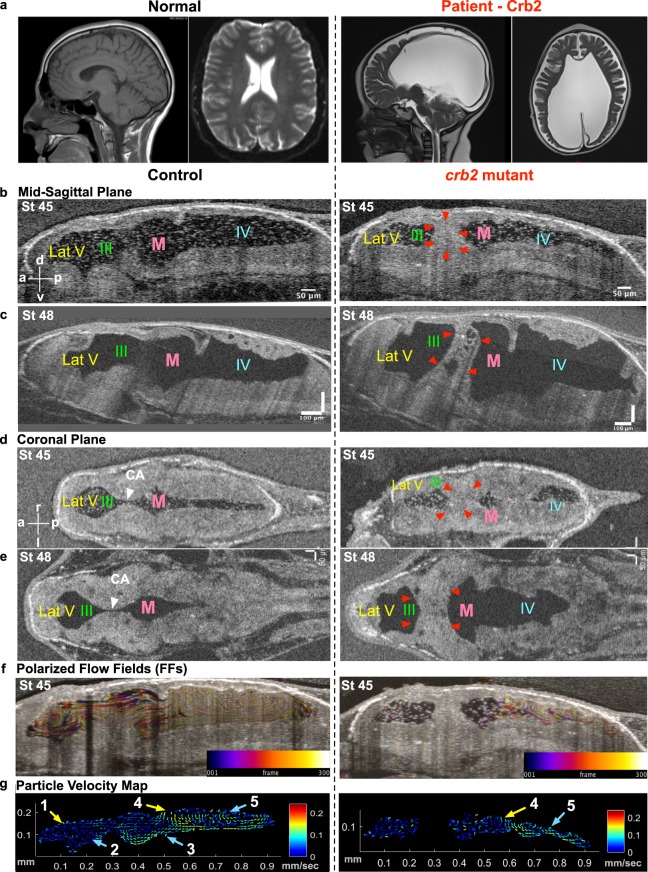
Hydrocephaly phenotype with CRB2 mutations. (**a**) CT scan of a normal brain and patient brain, with enlargement of the brain ventricles. (**b**) Mid-sagittal view (**d**) Coronal view using OCT shows reduction in ventricular size along with CA stenosis in *crb2* F0 CRISPR mutant tadpole brain at stage 45 as compared to controls. (**c**) Mid-sagittal view (**e**) Coronal view of control and *crb2* F0 CRISPR mutant tadpole raised to stage 48 shows enlargement of the ventricles as compared to controls. This result was seen in 3 animals that survived to this later stage. (White arrow head – CA, red arrowheads – stenosis of CA). (**f**) Stage 45 tadpole particle tracking shows the impairment of the flow fields in the lateral 3^rd^ and midbrain ventricles, but normal flow in the fourth ventricle for the *crb2* F0 CRISPR mutant as compared to the control. (**g**) 2D Particle Velocity Map shows loss of FFs 1-3 and intact FFs 4-5. (Lat-V: lateral ventricle, III: 3^rd^ ventricle, M: Midbrain ventricle, IV: 4^th^ ventricle).

We next used our *Xenopus* system to test a CH gene discovered in our CH cohort. Briefly, we evaluated a child born by cesarean section for macrocephaly, secondary to prenatally diagnosed severe, bilateral cerebral ventriculomegaly (Fig. 5a), to a woman with a previous history of recurrent pregnancy loss. At birth, the infant’s head circumference was ten weeks advanced of gestational age (46 centimeters; >99%ile for age), and the patient underwent uncomplicated ventriculo-peritoneal shunt placement on the first day of life (Fig. 5a).

Prenatal and postnatal chromosome analysis, microarray, and a comprehensive genetic panel for craniofacial syndromes and *L1CAM* X-linked hydrocephalus were negative, and the patient was subsequently referred to our institution for whole exome sequencing (WES). We identified compound heterozygous mutations: c.1062delA (p.Thr354fs) and c.2400C>G (p.Asn800Lys) in Crumbs homolog 2 (*CRB2*), a gene involved in ciliogenesis^33–35^ and previously implicated in a rare Mendelian syndrome of prenatal onset ventriculomegaly and steroid-resistant nephropathy (OMIM#609720)^33,36,37^. Sanger sequencing of PCR amplicons confirmed p.Thr354fs and p.Asn800Lys compound heterozygosity in the patient, and heterozygosity in each respective parent (primer sequences available on request).

To test if we could recapitulate a CH phenotype in *Xenopus*, we depleted *crb2* using F0 CRISPR and examined the ventricular system with OCT. At stage 45, *crb2* F0 CRISPR mutant tadpoles had smaller ventricles (Figs 5b,d, S3a,e–g). Both the dorsal-ventral height and the width of the ventricles were smaller although the anterior-posterior length was only mildly affected (Fig. S3e–g). 78% of the F0 CRISPR mutants showed stenosis of the aqueduct and only 12% showed an open connection, a phenotype recapitulated in a second non-overlapping sgRNA (Fig. S5a). Importantly, the flow of the lateral - 3^rd^ (FF1 and 2) and midbrain ventricles (FF3) was severely affected (Figs 5f,g, S2d, Movie 6), consistent with a role in cilia. Interestingly, we did not see any changes in the flow of the fourth ventricle (FF4 and FF5) (Fig. 5f,g, Movie 6), whether the flow in these posterior flow fields reflects near normal cilia function or choroid plexus flow remains to be determined. At stage 45, most of the *crb2* F0 CRISPR mutant tadpoles had aqueductal obstruction but no obvious hydrocephalus. However, at stage 48 (3 weeks post fertilization), the few tadpoles that survived to this stage developed signs of hydrocephalus and the cerebral aqueduct remained obstructed (Fig. 5c,e). To evaluate our F0 CRISPR strategy for *crb2*, we detected gene modification at the *crb2* locus and could rescue the *crb2* aqueductal stenosis with human CRB2 mRNA. We also detected a reduction in *crb2* signal by *in situ* hybridization especially in the ventricle (Fig. S5).

From these results, we conclude that depletion of *crb2* in *Xenopus* obstructs the communication between the lateral - 3^rd^ and midbrain - 4^th^ ventricles due to cerebral aqueduct stenosis, and this is associated, and likely due, to impairment of cilia-driven CSF flow in select populations of ependymal cells.

## Discussion

Congenital hydrocephalus (CH) is a common (1 per 1000 infants) and potentially lethal expansion of the CSF filled cerebral ventricles. Unfortunately, current treatment modalities are limited to morbid and antiquated brain surgery techniques^38^, in part due to our poor understanding of the interplay between ependymal cilia, embryonic CSF flow dynamics, and brain development. A recent NIH workshop identified a pressing need to establish animal models of CH in order to understand the genetic contribution to disease pathophysiology (Midbrain/Hindbrain Malformations and Hydrocephalus, 2014 NINDS). Our work addresses this need in two ways: 1) we develop a vertebrate animal model where the architecture of the brain ventricles can be visualized in real-time to map out an intact CSF flow network and 2) we use this model system to functionally assay the effect of CH genes on brain ventricular morphology, ependymal cilia function, and multi-ventricular CSF flow patterns.

CSF dynamics have been analyzed in various genetically tractable animal models including zebrafish, mice and *Xenopus*. As our goal is to model human disease, choosing an animal model as close to human is ideal. In isolated third ventricular explants, exogenously administered fluorescent particles can detect cilia-dependent flow^15^. However, these explant preparations cannot reproduce the three-dimensional CSF flow patterns in intact ventricular systems. Alternatively, CSF flow has been documented in zebrafish by injecting contrast reagents into the ventricular system^39^. However, *Xenopus* has certain evolutionary advantages for studying CH including lateral ventricles which the teleost lack and a neurulation process which is more similar between humans and *Xenopus* compared to zebrafish. In addition, the speed and low cost of making *Xenopus* models of CH makes *Xenopus* ideal for functionally testing CH candidate genes especially compared to the high cost of mouse. Previous work in *Xenopus* has identified CSF flow in a portion of the brain^40,41^. Miskevich in his work showed that fluorescent microspheres introduced to the ventricles with microinjection can be tracked using high speed camera in Albino *Xenopus leavis* tadpoles. He observed different velocities in different compartments of the brain which aligns with our current findings, yet this work was limited to visible parts of the ventricular system and didn’t show a global circulatory pattern^41^. Work by Hagencloher *et al*. showed that cilia were absent in *foxj1* morphants, causing impaired CSF flow and fourth ventricle hydrocephalus in tadpole-stage embryos. This study also tracked fluorescent microspheres in the fourth ventricle using high speed imaging^40^. In our work, we comprehensively describe CSF flow through the entire ventricular system with its distinct polarity. We also reveal that the nature of ciliary gene affected can lead to distinct phenotypes and demonstrate its utility as a hydrocephalus model. When paired with OCT imaging, *Xenopus* provides a genetically tractable system where brain development, ventricular patterning, ependymal cilia function, and CSF microcirculatory dynamics can be simultaneously investigated and genetically perturbed in a non-destructive, label-free fashion. The candidate genes which show disease causality in our model, can be further studied using previously established methods to get at the underlying mechanism for gene function.

Using our *Xenopus*-OCT platform, we can detect the global CSF flow network mapped to CNS structures. We detect five discrete flow fields nested within three ventricles. Interestingly, unlike other ciliated surfaces that are polarized along a single axis, the CSF flow fields are location- and context-specific, suggesting patterning and polarization of the underlying ependymal cilia. For example, the telencephalic lateral ventricle has two discrete ciliary surfaces polarized in opposite directions generating a flow that unites in the center and forms a flow vector oriented towards the ventrally located optical tectum. Future work will define the cilia polarity and beating characteristics that are location specific to create these unique flow fields as well as the analysis of the 3D flow. Analyzing the subcellular architecture required for cilia polarization will prove fruitful for understanding how these flow fields are generated and how they change in disease states.

In analyzing flow fields with particle tracking, the presence of endogenous particles was fortuitous. By tracking particles, we can compute velocities in real time since the number and speed of these particles can be resolved with OCT imaging. Based on this data, we calculated average velocity maps to compare regional changes in velocities among the control and perturbed embryos. The average velocity maps can quantify mean velocity changes across multiple samples and highlight the dramatic changes in CSF flow across conditions (Fig. S2).

We also counted the number of particles within the ventricles and created heat maps to indicate particle concentration. Similar to averaged velocity maps, we formed averaged particle maps and analyzed the particle distribution across multiple embryos of a given condition. In wild types, we saw an even distribution of these particles across the ventricles, despite differentially polarized flow fields. The mean particle count per mm^2^ also remained constant over time (Fig. S2a). In *c21orf59* morphants, where cilia motility was diminished and the tadpoles developed aqueductal stenosis further limiting ventricular mixing, we observed a slightly reduced total particle counts but surprisingly a relatively even distribution (Fig. S2a). In contrast, *foxj1* morphants had significantly more particles even after adjusting for the larger ventricular volume but again the particles were evenly distributed (Fig. S2b). On the other hand, *l1cam* F0 CRISPR mutants had an uneven particle distribution where most of the particles were confined to the third and fourth ventricle, much less in the lateral ventricle. The change in distribution was not a result of a loss of particles; in fact, *l1cam* F0 CRISPR mutants had slightly more particles than wildtype controls (Fig. S2c). Surprisingly, *crb2* F0 CRISPR mutants had the near opposite. Compared to wildtype controls, crb2 F0 CRISPR mutants had fewer particles that were confined to the lateral ventricles (Fig. S2d). These observations clearly suggest that alterations in CSF circulation, ependymal patterning and/or ventricular patterning may result in differential mixing or maldistribution of potential CSF derived factors. Whether these particles are vesicles or some other physiologically relevant structures and their importance in ventricle patterning and hydrocephalus pathogenesis is worth pursuing in the future.

**Figure 6:**
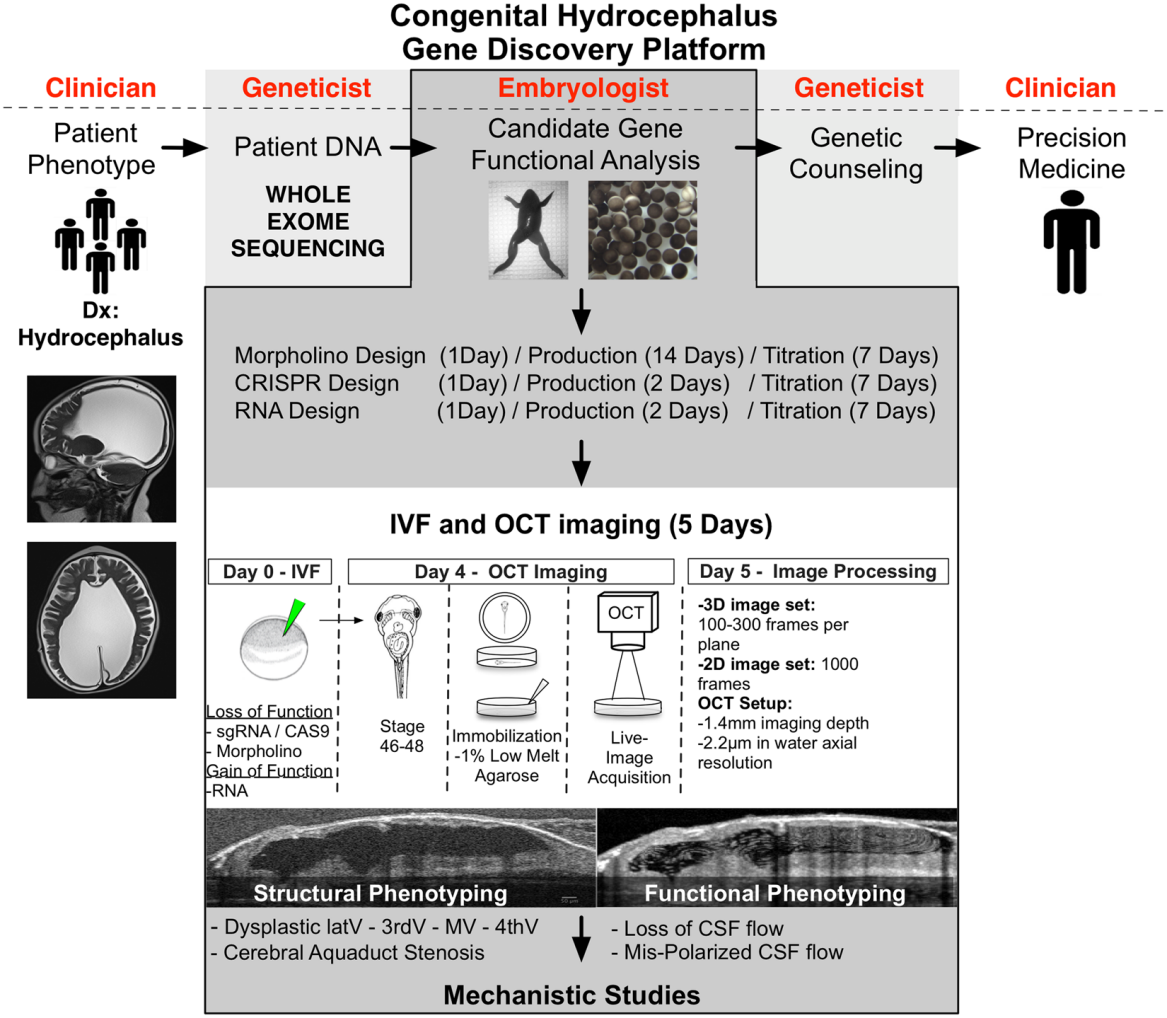
Schematic representation of the gene discovery platform for screening novel developmental hydrocephaly genes using *Xenopus* and OCT as a model system.

The genetic basis of hydrocephalus is at the early stages of gene discovery with only a handful of genes identified so far^38^. The most daunting problem for genetic analysis is high locus heterogeneity. To date only four *bona fide* genes, *L1CAM*, *AP1S2*, *MPDZ* and *CCDC88C* have been directly shown to be causal. With the plummeting cost in gene sequencing, we readily detect gene variants in our patients^42^. However, the high locus heterogeneity complicates assigning disease causality and is likely to identify a number of genes whose mechanism of hydrocephalus pathogenesis is unknown. Therefore, rapid functional testing of these variants in model systems remains a crucial step. Recently, an analysis of 27 families with recurrent hydrocephalus demonstrated a remarkably high contribution of recessive mutations to congenital hydrocephalus^42^. *Xenopus* is an ideal system to evaluate the genetic and physiological impact of these potential candidate genes inexpensively and in a short period of time (F0 CRISPR mutants can be generated and phenotyped in 5 days)^28^. If the candidate genes recapitulate the hydrocephalus phenotype in our *Xenopus* model, this data bolsters the evidence for disease relevance and is also crucial to provide mechanistic insight about hydrocephalus pathophysiology. Our results with L1CAM and CRB2 indicate that *Xenopus* is efficient and robust for CH modeling. As WES identifies novel CH candidate genes, our *Xenopus* model may serve as a robust system to validate implicated genes, as well as to use for drug screening to develop of targeted therapeutics for specific CH subtypes (Fig. 6).

## Materials and Methods

### *Xenopus* tropicalis

*Xenopus tropicalis* were housed and cared for in our aquatics facility according to established protocols that were approved by the Yale Institutional Animal Care and Use Committee (IACUC) in accordance with NIH guidelines. Female and male mature *Xenopus tropicalis* were obtained from National *Xenopus* Resource.

### Subjects and samples

All study procedures and protocols comply with Yale University’s Human Investigation Committee and Human Research Protection Program. The study has been approved by Yale Human Investigation Committee (HIC protocol #1602017144) and by Yale IRB board. Written informed consent for genetic studies was obtained from all participants. Our patient and her participating family members provided buccal swab samples (Isohelix SK-2S DNA buccal swab kits), medical records, radiological imaging studies, operative reports, and congenital hydrocephalus phenotype data.

### Microinjections

*Xenopus* embryo *in vitro* fertilization and microinjections were performed as per standard protocols^24^. Embryos were injected with CRISPR sgRNA and mRNA at the one cell stage. For microinjections, borosilicate glass needles were calibrated to inject 1.6 ng Cas9 protein with 200 pg of the CRISPR sgRNA and a fluorescent tracer Alexa488 (Invitrogen). The target sequence for the *l1cam* CRISPRs are GGGGACTGCTCAGTAATCAC and GGTGGCTGTGCCCAGATCAT and for *crb2* CRISPRs are GGCAGCCCTGTCAGAATGGA and GGTCTCCATCATAGCCCGCT. To make F0 CRISPR mutants, we generated sgRNAs using the EnGen sgRNA synthesis kit (NEB) following the manufacturer’s instructions. Full length human WT *CRB2* RNA construct was a kind gift from Dr. Nishimura, Shiga University of Medical Science. It was sub-cloned in the standard *Xenopus* vector, pCS2 and capped mRNA was synthesized *in vitro* using the mMessage machine kit (Ambion) as per the manufacturer’s instructions. We injected 12.5 and 25 pg wild type human *CRB2* RNA for rescue of *crb2* F0 CRISPR mutants. The following morpholino oligonucleotide was injected at 4-cell stage: *foxj1* translation blocking (8 ng/embryo 5′-ATGCTGCTGTTGGGCATCTTCCTCC-3′) and the *c21orf59* translation blocking (8 ng/embryo 5′-CCTTCTTAACGTGTAAGCGCACCAT-3′). Post-injections, embryos were incubated in 1/9X MR supplemented with 50 μg/ml of gentamycin at 25 °C. Injections were confirmed by fluorescent lineage tracing with a Zeiss Lumar fluorescence stereomicroscope at stage 28 and tadpoles further raised at until stage 45.

### F0 CRISPR mutant genotyping

Genomic DNA was extracted from each embryo using a DNeasy Blood & Tissue Kit (Qiagen). The target regions were amplified by PCR with 35 cycles to facilitate the heteroduplex formation using Phusion® High-Fidelity DNA Polymerase (NEB). PCR products were then annealed and digested with T7 Endonuclease I (NEB) as per the manufacturer’s instructions and run on a 2% agarose gel to determine genome targeting efficiency.

### Immunofluorescence

We fixed control and mutant tadpoles at stage 45. We then dissected the brains and performed immunofluorescence of L1CAM (Abcam – ab24345) at 1:500. Alexa 488 (1:500) was used as a secondary antibody. Alexa 647 phalloidin (1:40) was used for actin staining. Embryos were mounted in Pro-Long Gold (Invitrogen) before imaging. Images were captured using a Zeiss 710 confocal microscope. Images were processed in Fiji, Image J, or Adobe Photoshop. Final figures were made in Adobe illustrator.

### RNA *in situ* hybridization

*X. tropicalis* embryos were collected at stages 28 and stage 45. *in-situ* hybridization was performed according to standard protocols^43^. Additional probe information is available upon request.

### Optic Coherence Tomography and Particle Tracking

Stage 45 (post-fertilization Day 4) tadpoles were immobilized in 2.5 ml of 1% low melt agar in a 35 mm diameter −10 mm height polystyrene petri dish. The tadpoles were aligned such that the dorsal side of the body was exposed en face to the OCT imaging field. We used a Thorlabs Ganymede 900 nm spectral domain-OCT Imaging System, which allows for 1.4 mm imaging depth in air + water −2.2 µm Axial Resolution in water. We obtained 2D cross sectional and 3D images by scanning at 36 kHz (36000 A-scans per second) with maximal 101 dB sensitivity. Measurements obtained from Thorlabs software ThorImageOCT version 4.4.6, analyzed using Prism7 statistical software. The data was analyzed to check if it fits normal distribution and an independent-samples t-test was conducted to compare different ventricular measurements for control and mutant embryos.

Flow velocimetry was performed in four steps: (1) anisotropy removal, (2) particle tracking, (3) region of interest selection, (4) particle displacement averaging to yield flow velocities. Further, we analyzed spatial particle distribution and present it as heatmaps (5). Next, (6) flow maps and heatmaps across multiple animals were created. The ventricle volume and 3D shape were also examined (7).

All image and data processing was performed using Matlab (The MathWorks, Natick, Massachusetts, USA, Version R2017b) and ImageJ (National Institutes of Health, USA, Version 1.5.1 u).

#### Anisotropy Removal

Images from OCT are usually not stored as isotropic images (images of equal physical height and length). Various settings and factors during acquisition dictate the aspect ratio of a pixel (or voxel respectively), such as refractive index and beam movement. Using this information, width and height of each pixel was adjusted to have square aspect ratio.

#### Particle Tracking

Particle tracking was performed using Mosaic particle tracker^44^ plugin build in ImageJ^45^. The plugin allows two-dimensional tracking for automated detection and analysis of particle trajectories. It is self-initializing, discriminates spurious detections, can handle temporary occlusion as well as particle appearance and disappearance from the image region to some degree.

#### Region of Interest Selection

In order to exclude any particles tracked outside the ventricle, the region of interest (ROI) was segmented. This was done manually using the MATLAB tool CROIEditor. All particle tracking data outside of the ROI was removed before any analysis.

#### Flow Velocity

Particle trajectories were averaged across all frames of each sample to yield mean flow at each position. For visualization purposes, the number of vectors in the flow velocity plots was reduced to 10-by-10 pixel non-overlapping windows. Within each window, the average flow was calculated. After averaging, magnitudes above the 97th percentile were considered outliers caused by incorrect particle tracking and therefore removed.

#### Particle Quantity and Particle Distribution Heatmaps

The number of particles in a given sample was calculated as the mean number of particles in each frame (extracted from particle tracking information) and was normalized using the sample’s ROI area. Where particle tracking yielded a high rate of false negatives (this was limited to *foxj1* morphants where particle counts were significantly high), we shifted to a single-image-based approach consisting of H-dome transform^46^ and subsequent particle detection using boundary detection^47^.

Spatial distribution of particles is conveyed through heatmaps which are also based on the particle tracking information. They show the spatial particle density averaged over all frames of a video.

#### Cross-Animal Flow Fields and Heatmaps

Averaged flow maps and particle distribution heatmaps of animals of a group were created by first matching the ROIs to each other using Procrustes analysis^48^. Ventricle outlines were used as input to the Procrustes function, which returns rotation, translation and scaling to be applied to a set of points (describing the ventricle outline/ROI) in order to transform them such that they match another set of input points (another ventricle outline/ROI) as closely as possible (based on minimizing the sum of squared errors). This resulting transformation information was applied to corresponding flow vectors/heatmaps. Finally, all transformed flow vectors/heatmaps of a group were averaged.

#### Ventricle Volume Measurement and Plot

Employing a region growing algorithm^49^ we extracted 3D representations of the ventricle and its volume. Seed points for the region growing algorithm were chosen manually. High particle density caused unsatisfactory performance of this approach. In these cases, manual segmentation of several slices was performed and the full volume was interpolated. Validation of this method was conducted by comparing volume measurements using the above described automatic approach with the manual approach on 12 different samples. Volume measurements of the automatic and manual approach showed a mean error of 0.005602574 mm^3^ and a standard deviation of the error of 0.006480865 mm^3^. This is based on 11–12 slices of the z-stack with manual ventricle segmentation.

### Whole-exome sequencing and variant calling

DNA was obtained from buccal swab samples in accordance with manufacturer protocol. Whole-exome sequencing was performed using the Nimblegen SeqxCap EZ MedExome Target Enrichment Kit (Roche Sequencing, Pleasanton, CA, USA) followed by 99 base paired-end sequencing on the Illumina HiSeq 2000 instrument at the Yale Center for Genome Analysis as previously described^50^. Sequence reads were mapped and aligned to the GRCh38/hg19 human reference genome using Burrows-Wheeler Aligner-MEM^51^ and further processed using Genome Analysis Toolkit (GATK) base quality score recalibration^52^, indel realignment, duplication marking and removal, and base quality score recalibration in accordance with GATK Best Practices recommendations^53,54^. Single nucleotide variants and small indels were called using GATK Haplotype Caller and annotated using ANNOVAR^55^, DbSNP^56^, 1000 Genomes^57^, NHLBI exome variant server^58^, and gnomAD and ExAC databases^59^.

### Kinship analysis

Pedigree information and participant relationships were confirmed utilizing pairwise PLINK identity-by-descent calculation^60^.

### De novo and Inherited (Dominant/Recessive) variant analysis

The Bayesian framework TrioDeNovo was used to call *de novo* mutations in the parent-offspring trio, consisting of our patient and her biological parents^61^. *De novo* candidates were filtered based on the following criteria of: (1) possessing a minor allele frequency (MAF)  ≤ 5 × 10^−3^ in ExAC, (2) having a GATK variant quality score recalibration (VQSR) of ‘pass’, (3) meeting a minimum sequencing depth of 8 reads in the proband and 12 reads in each parent, (4) having a genotype quality (GQ) score ≥20 and alternate allele ratio ≥40%, (5) earning a TrioDeNovo data quality (DQ) score ≥7, and (6) being an exonic or canonical splice-site variant.

Transmitted variants were filtered similarly, with dominant variants meeting the following criteria of rareness and quality: (1) MAF ≤ 2 × 10^−5^ in ExAC, (2) GQ ≥ 20 and alternate allele ratio ≥40%, (3) GATK VQSR of ‘pass’, and (4) minimum sequencing depth of 8 reads in each participant. Recessive variants were filtered for rare (MAF ≤ 10^−3^ in ExAC) homozygous and compound heterozygous mutations that met read quality criteria as above (GQ ≥ 20, alternate allele ratio ≥40%, ‘pass’ GATK VQSR, and minimum sequencing depth ≥8).

We predicted the impact of nonsynonymous single nucleotide variants on protein function using the MetaSVM algorithm^62^, identifying mutations with rank scores greater than 0.83357 as deleterious (‘D-mis’). Only D-Mis and loss-of function mutations (nonsense, canonical splice-site, and frameshift indels) were considered potentially damaging to protein function.

All *de* novo and transmitted calls were verified by *in silico* visualization of aligned reads using the Integrative Genomics Viewer (IGV)^63^ and BLAT search^64^. Resultant *de novo* and compound heterozygous calls were then verified in the patient and both parents by direct Sanger sequencing of PCR amplicons containing the mutation. A compound heterozygous mutations: c.1062delA (p.Thr354fs) and c.2400C>G (p.Asn800Lys) was identified in Crumbs homolog 2 (*CRB2*), The maternally-inherited p.Thr354fs mutation is a novel, frameshift deletion not reported in large, reference databases (gnomAD, ExAC, 1000 Genomes Project, NHLBI). The second, paternally inherited p.Asn800Lys mutation occurs in a highly conserved residue and is extremely rare in the general population (MAF of 1.4 × 10^−4^ in the ExAC database). Sparse case reports demonstrate pathogenicity of this substitution in compound heterozygous state with other *CRB2* mutations; interestingly, all previously reported patients harboring the p.Asn800Lys mutation were of Ashkenazi Jewish descent^33,36^. Analysis from 128 genomes of Ashkenazi Jewish ancestry identified two *CRB2*-p.Asn800Lys heterozygotes, demonstrating a 1:64 carrier frequency in the population^33,34^.

### Whole-Exome Sequencing

The average depth of coverage of the whole-exome sequencing data was 41.2×, with greater than 8x coverage in 96.40% of the target region for exome capture. The pedigree suggested a sporadic or autosomal recessive mode of inheritance, so compound heterozygous, homozygous, and *de novo* variants were prioritized. Variants were filtered for predicted deleteriousness using a series of *in silico* prediction algorithms, and excluded if above standard rare minor allele frequency thresholds in ExAC and other established databases.

## Quantification and Statistical Analysis

The data was analyzed to check if it fits normal distribution. Measurements via OCT Thorlabs software represented by the mean value ± SEM for each group. Two-tailed Student’s t test analysis or a chi-square test of independence was used to examine all significant differences between groups as indicated in the figure legends. Significance was determined when the p value is lower than 0.05.

## Supplementary information

Supplementary Data

Movie 1

Movie 2

Movie 3

Movie 4

Movie 5

Movie 6

Movie 7
