# Author Correction: Visualizing flow in an intact CSF network using optical coherence tomography: implications for human congenital hydrocephalus

**DOI:** 10.1038/s41598-020-59301-y

**Published:** 2020-02-12

**Authors:** Priya Date, Pascal Ackermann, Charuta Furey, Ina Berenice Fink, Stephan Jonas, Mustafa K. Khokha, Kristopher T. Kahle, Engin Deniz

**Affiliations:** 1 Pediatric Genomics Discovery Program, Yale University School of Medicine, 333 Cedar Street, New Haven, CT 06510 USA; 2 Department of Pediatrics, Yale University School of Medicine, 333 Cedar Street, New Haven, CT 06510 USA; 3 Department of Medical Informatics, Uniklinik RWTH Aachen, Pauwelsstr 30, 52074 Aachen, Germany; 4 Department of Genetics, Yale University School of Medicine, 333 Cedar Street, New Haven, CT 06510 USA; 5 Department of Informatics, Technical University of Munich, Munich, Germany; 6 Department of Neurosurgery and Cellular & Molecular Physiology, and Centers for Mendelian Genomics, 333 Cedar Street, New Haven, CT 06510 USA

Correction to: *Scientific Reports* 10.1038/s41598-019-42549-4, published online 17 April 2019

In this Article, the Supplementary Information movies are incorrectly referenced. As a result, in the Results section under the subheading ‘Loss of cilia motility or biogenesis causes discrete ventricular phenotypes’,

“These tadpoles demonstrated no or very slow CSF flow velocities as detected by OCT imaging (Fig. 3b,c).”

should read:

“These tadpoles demonstrated no or very slow CSF flow velocities as detected by OCT imaging (Fig. 3b,c – Movie 4).”

Additionally, in the same section,

“We observed a dramatic loss of flow throughout the ventricles, but unlike the *c21orf59* knockdown, the ventricles were significantly larger, resembling communicating hydrocephalus (Figs. 3d,e, S2b, S3a).”

should read:

“We observed a dramatic loss of flow throughout the ventricles, but unlike the *c21orf59* knockdown, the ventricles were significantly larger, resembling communicating hydrocephalus (Figs. 3d,e, S2b,S3a – Movie 5).”

In the Results section under the subheading ‘Loss of *L1CAM* function in *Xenopus* tadpoles causes aqueductal stenosis’,

“Next, we investigated the ventricular CSF flow and mapped FF1 to FF5, which appeared intact (Figs. 4e,f, S2c, Movie 5).”

should read:

“Next, we investigated the ventricular CSF flow and mapped FF1 to FF5, which appeared intact (Figs. 4e,f, S2c, Movie 6).”

In the Results section under the subheading ‘*CRB2* loss-of-function leads to cerebral aqueduct stenosis and ventriculomegaly in humans and frogs’,

“Importantly, the flow of the lateral – 3^rd^ (FF1 and 2) and midbrain ventricles (FF3) was severely affected (Figs. 5f,g, S2d, Movie 6), consistent with a role in cilia. Interestingly, we did not see any changes in the flow of the fourth ventricle (FF4 and FF5) (Fig. 5f,g, Movie 6), whether the flow in these posterior flow fields reflects near normal cilia function or choroid plexus flow remains to be determined.”

should read:

“Importantly, the flow of the lateral – 3^rd^ (FF1 and 2) and midbrain ventricles (FF3) was severely affected (Figs. 5f,g, S2d, Movie 7), consistent with a role in cilia. Interestingly, we did not see any changes in the flow of the fourth ventricle (FF4 and FF5) (Fig. 5f,g, Movie 7), whether the flow in these posterior flow fields reflects near normal cilia function or choroid plexus flow remains to be determined.”

