# Supplementary material for: Visualizing flow in an intact CSF network using optical coherence tomography: implications for human congenital hydrocephalus
Source: Sci Rep. 2019 Apr 17;9:6196. doi: 10.1038/s41598-019-42549-4 (PMC6470164; doi:10.1038/s41598-019-42549-4)
Supplement: Supplementary file 1 — Supplementary Data [file 41598_2019_42549_MOESM1_ESM.pdf]

Visualizing flow in an intact CSF network using optical coherence tomography: implications for human congenital hydrocephalus

Priya Date[1,2>], Pascal Ackermann[2,3>], Charuta Furey[4,6], Ina Berenice Fink[3], Stephan Jonas[5], Mustafa K. Khokha[1,2,4], Kristopher T. Kahle[2,4,6*] and Engin Deniz[1,2,*]

[1]Pediatric Genomics Discovery Program, Yale University School of Medicine, 333 Cedar Street, New Haven, CT 06510, USA

[2]Department of Pediatrics, Yale University School of Medicine, 333 Cedar Street, New Haven, CT 06510, USA

[3] Department of Medical Informatics, Uniklinik RWTH Aachen, Pauwelsstr. 30, 52074 Aachen, Germany

[4]Department of Genetics, Yale University School of Medicine, 333 Cedar Street, New Haven, CT 06510, USA

[5]Department of Informatics, Technical University of Munich, Munich, Germany

[6]Department of Neurosurgery and Cellular & Molecular Physiology, and Centers for Mendelian Genomics, 333 Cedar Street, New Haven, CT 06510, USA

>equal contribution

*Corresponding authors:
engin.deniz@yale.edu
kristopher.kahle@yale.edu

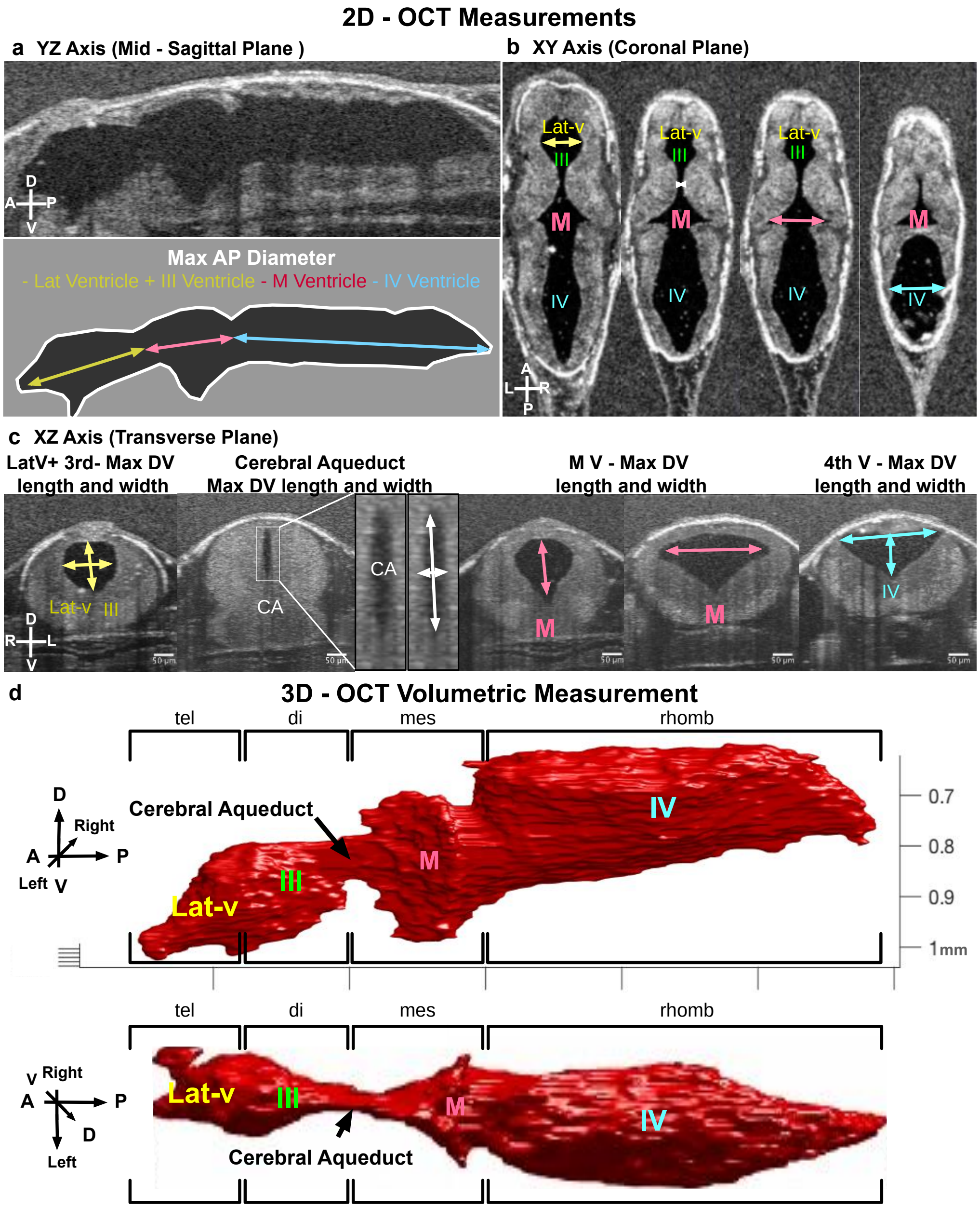

Supplemental Figure 1. Measurements by OCT of the *Xenopus* tadpole ventricular system.
(a) Mid-sagittal view (YZ axis): The dorsal-ventral (DV) plane along the anterior-posterior (AP) axis of the ventricular system. The AP length measurement positions are shown for the lateral ventricle and 3rd ventricle in yellow, midbrain ventricle in purple and 4th in blue.
(b) Coronal view (XY axis): The top view of the ventricular system is visualized using the 3D acquisition. The width of each ventricle can be measured in this view along with the CA width.
(c) Transverse view (XZ axis): DV height and Left-Right (LR) width for each of the ventricle is shown when moving from anterior to the posterior of the animal. The Lateral and 3rd ventricle is shown at its widest and deepest section followed by the height and width of the Cerebral Aqueduct (CA). The midbrain ventricle is deepest anteriorly and becomes wider as it merges into the 4th ventricle which is shown as the last image.
(d) 3D rendering of the ventricular system presented on sagittal and coronal plane.

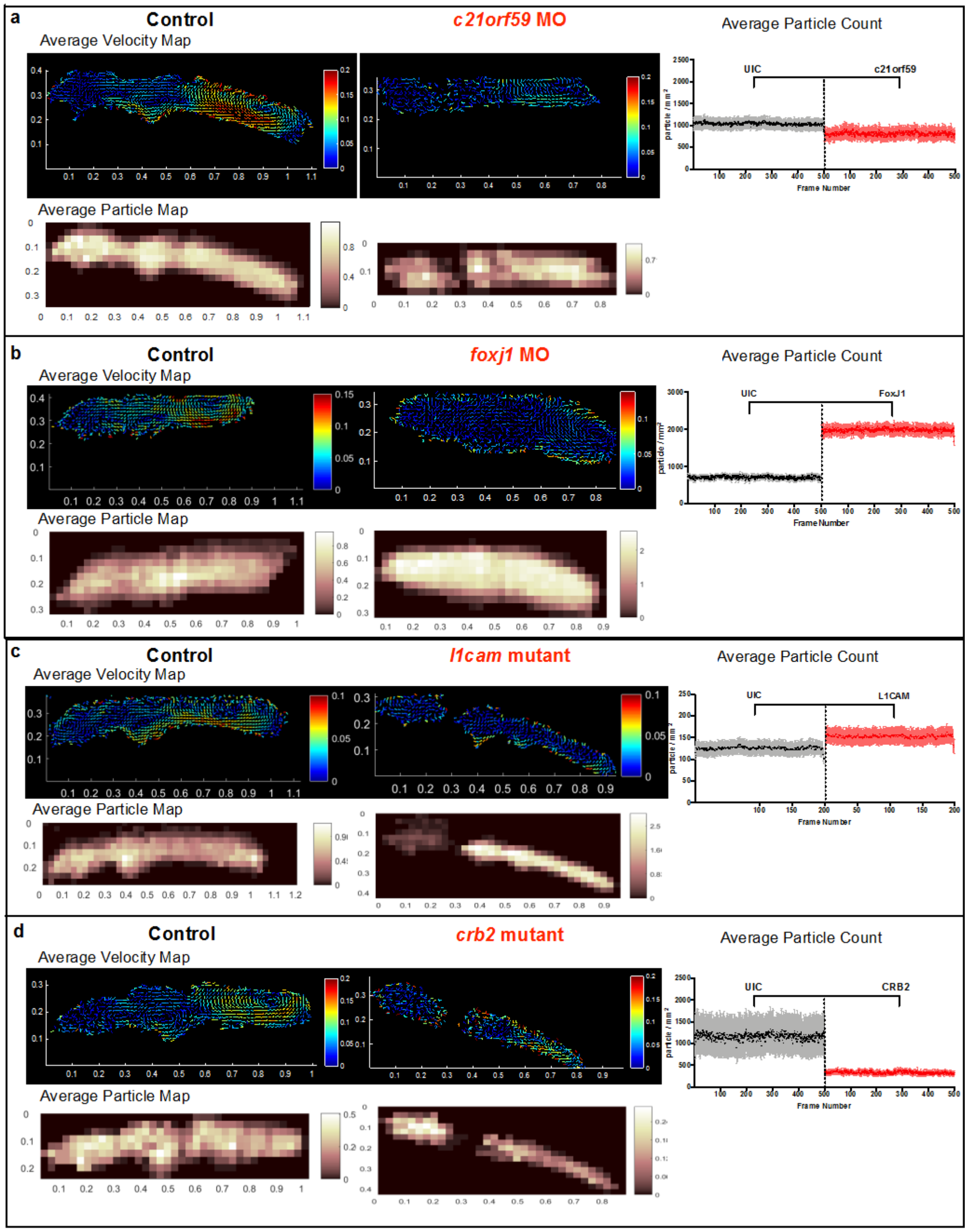

Supplemental Figure 2. Averaged flow maps and average particle distribution heatmaps were created by Procrustes analysis (see methods for details).

(a) Average velocity map for *c21orf 59* morphants show slower particle velocities. Average particle maps show a globally even presence of particles across the ventricles. Total particle counts were lower in morphants.

(b) Average velocity map for *foxj1* morphants show slower particle velocities. Average particle maps show uniformly distributed particles across the ventricles. Total particle counts were significantly higher in morphants.

(c) Average velocity map for *l1cam* F0 CRISPR mutants show slightly slower particle velocities but intact flow fields. Average particle maps show an uneven distribution of particles, more present within the midbrain and 4th ventricle compared to the lateral and 3rd ventricle. Total particle counts were higher in mutants.

(d) Average velocity map for *crb2* F0 CRISPR mutants show slower particle velocities except in distal 4th ventricle. Average particle maps show an uneven particle distribution, with more present within the lateral ventricle. Total particle counts were very low in mutants.

(control n=11, *c21orf59* MO n=11, *foxj1* MO n=11, *l1cam* F0 CRISPR Mutant n=11, *crb2* F0 CRISPR Mutant n=11).

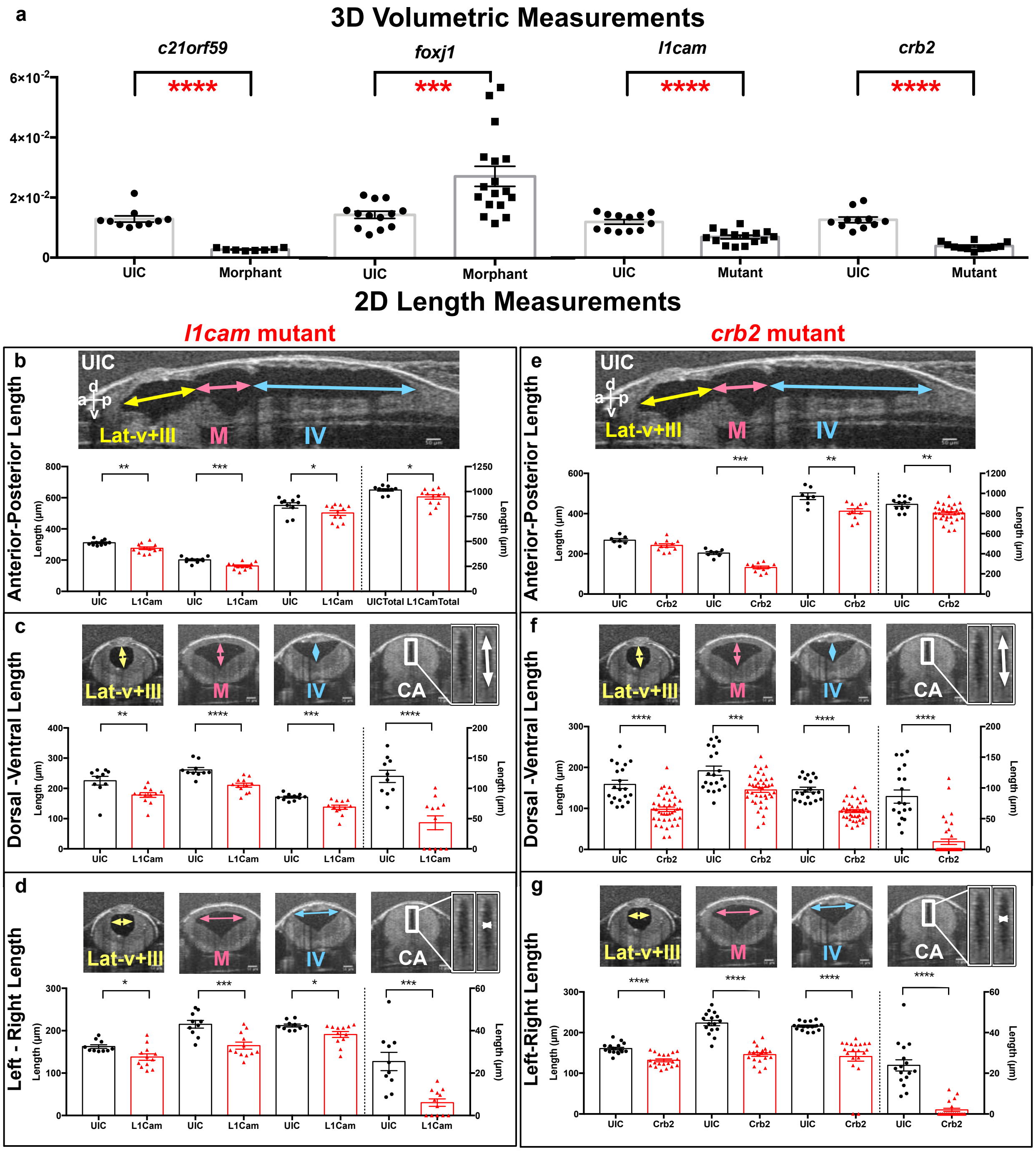

Supplemental Figure 3. Quantitative analysis of the size of different ventricles for control and mutant frogs using OCT.

(a) Comparison of 3D volumetric measurements for the ventricular system for *c21orf59* and *foxj1* morphants and *l1cam* and *crb2* F0 CRISPR mutants with their respective controls. (n=10-20). Schematic representation of position of 2D measurements taken on the control vs. *l1cam* (left column) and *crb2* (right column) F0 CRISPR mutant ventricles are shown at the start of each panel.

(b, e) Anterior-posterior length measurements: Mutants show reduced length for individual ventricles as well as of the entire ventricular system.

(c, f) Dorsal-ventral height measurements for each ventricle and CA.

(d, g) Left-right width for each individual ventricle and the CA. (Total: Total ventricular system length from the most anterior tip of the lateral ventricle to the most posterior tip of the 4th ventricle. *P<0.05, **p<0.01, ***p<0.001, ****p<0.0001. Bars indicate the mean value and each individual data point is shown (n=10-40).)

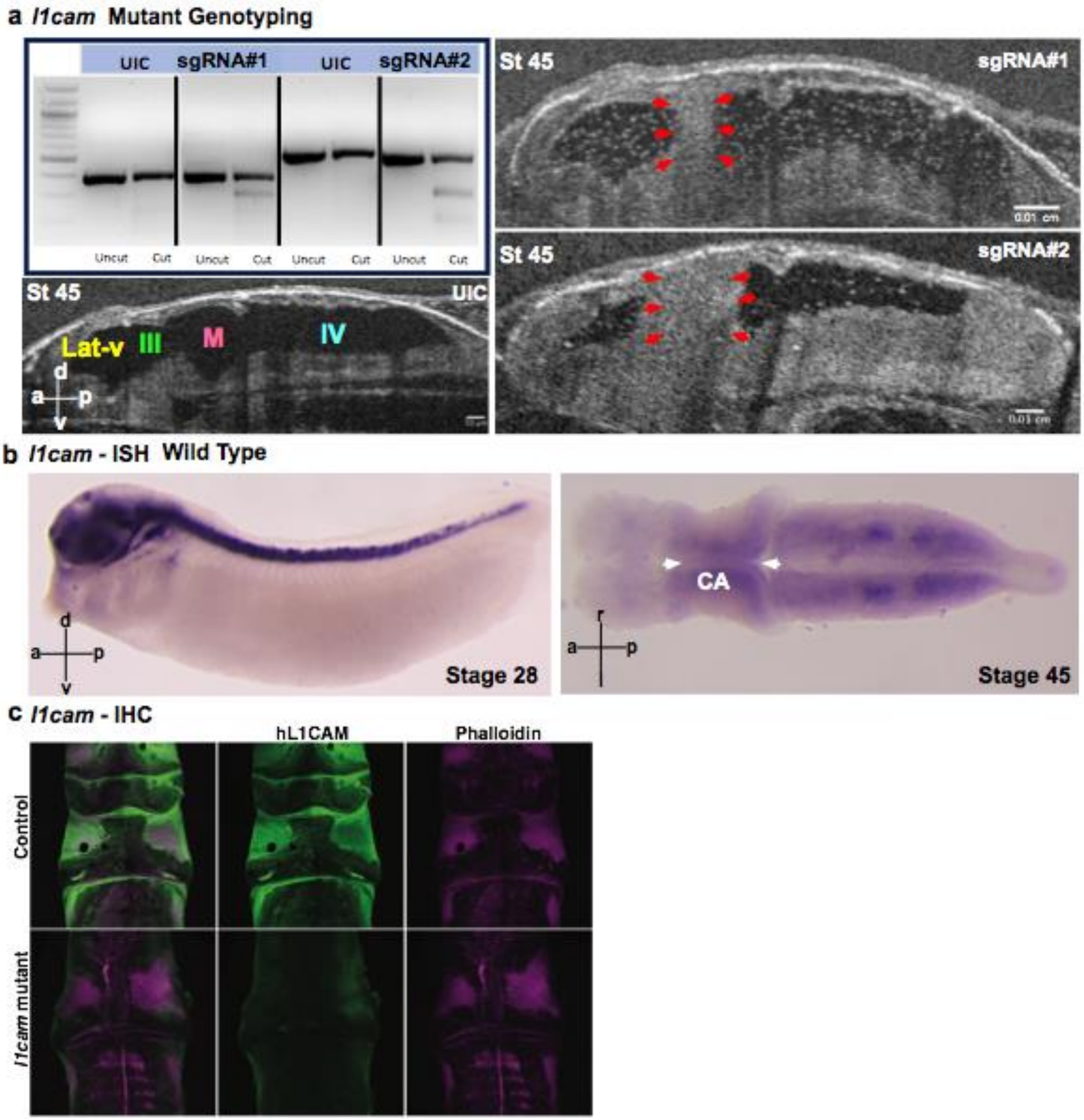

Supplemental Figure 4. Validation of *l1cam* F0 CRISPR mutant.

(a) DNA gel showing the PCR product for each *l1cam* F0 CRISPR mutant and control, before and after cutting with T7 endonuclease I. Mid sagittal OCT image of control and two independent *l1cam* F0 CRISPR mutants, the later showing stenosis of the cerebral aqueduct (red arrowheads).

(b) At stage 28 *l1cam* mRNA expression can be seen in the eye, brain, spinal cord and the notochord using *in-situ* hybridization. At stage 45 it is weakly expressed in forebrain but a strong expression is seen in the mid and hind brain region.

(c) Immuno-fluorescence against l1cam protein shows loss of the signal in mutant as compared to the control stage 45 brain.

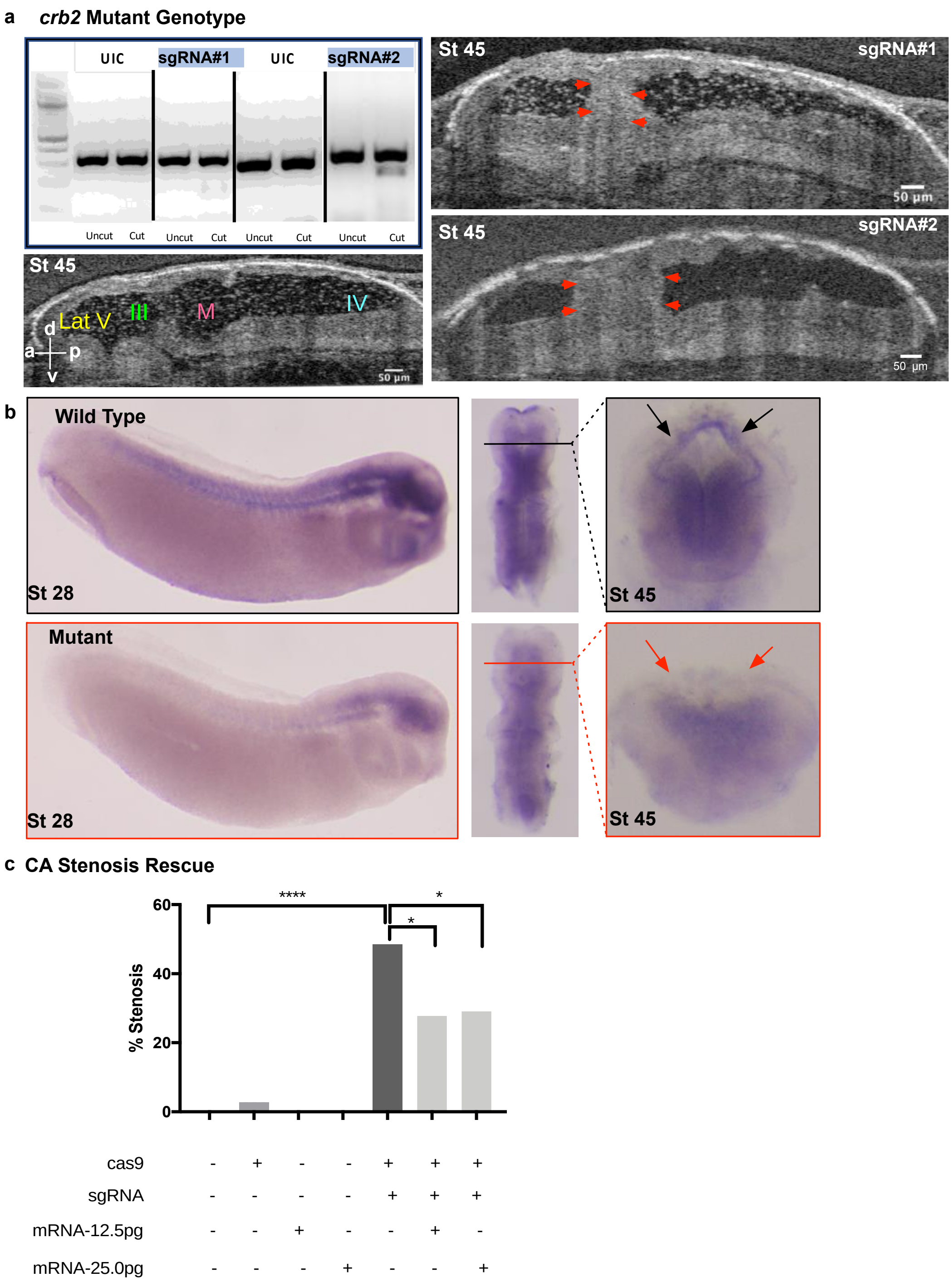

Supplemental Figure 5. Validation of *crb2* F0 CRISPR mutant.

(a) DNA gel showing the PCR product for each *crb2* F0 CRISPR mutant and control, before and after cutting with T7 endonuclease I. Mid sagittal OCT image of control and two independent *crb2* F0 CRISPR mutants, the later showing stenosis of the cerebral aqueduct (red arrowheads).

(b) At stage 28 *crb2* mRNA expression can be seen in the eye and brain with weaker expression in the spinal cord and notochord using *in-situ* hybridization (n=30). This expression is lost in the mutant animals (n=15). At stage 45, *crb2* mRNA is expressed in the whole brain, specifically in the ependymal lining of the ventricles (black arrows in the inset showing a cross section of the brain). This expression is reduced in the mutants (red arrows).

(c) Rescue experiment shows a reduction in the proportion of animals that get cerebral aqueduct stenosis when the *crb2*-sgRNA is co-injected with human WT *crb2* mRNA. (sample size for each treatment: control=30, cas9-only=36, sgRNA=66, 12.5pgmRNA=45, 25pgmRNA=55, 12.5pg_mRNA+sgRNA=72, 25pg_mRNA+sgRNA=55). A chi-square test of independence was performed to examine the relation between treatments. *P<0.05, ****p<0.0001.

(a) ***l1cam* Mutant Genotype - sgRNA#1 & 2**

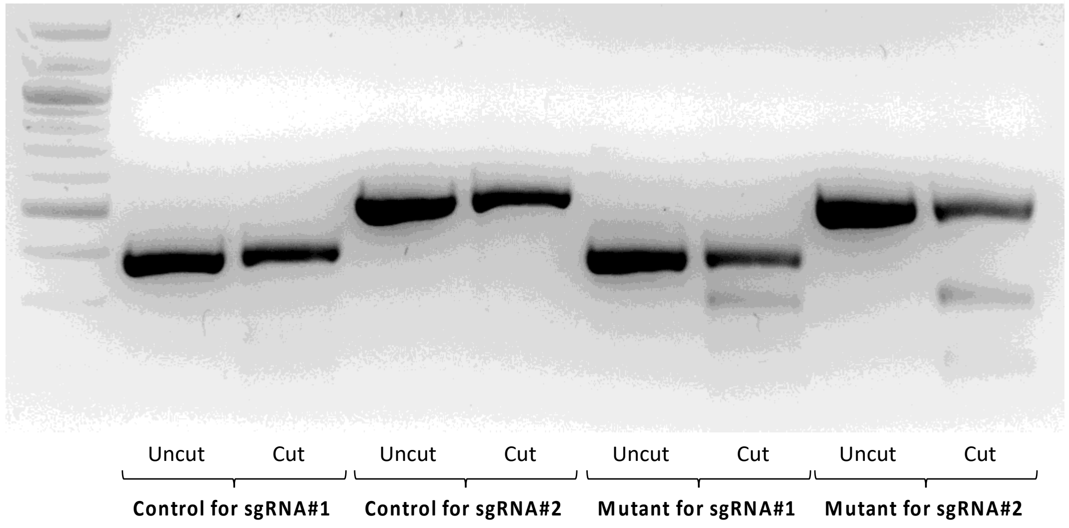

(b) ***crb2* Mutant Genotype - sgRNA#1**

***crb2* Mutant Genotype - sgRNA#2**

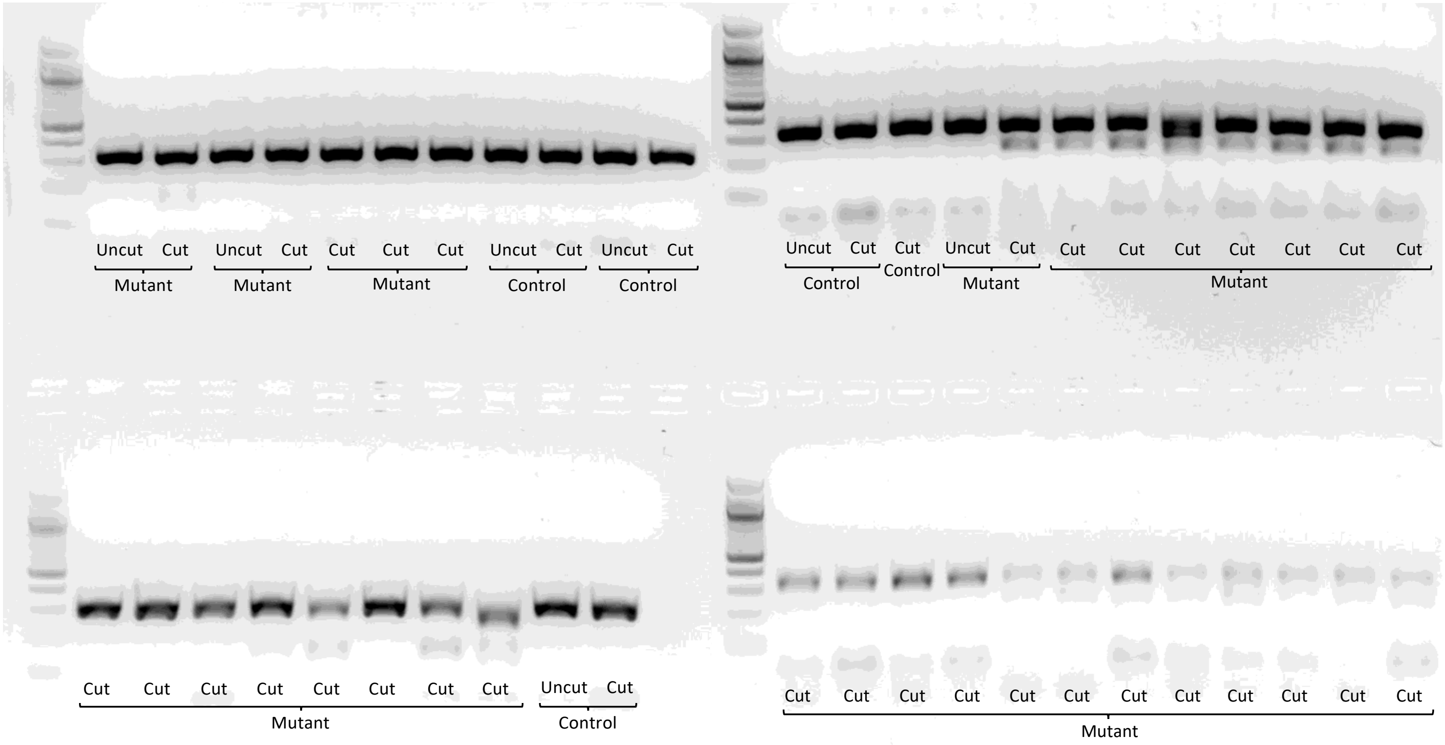

Supplemental Figure 6

(a) Full - DNA gel showing the PCR product for each *l1cam* F0 CRISPR mutant and control, before and after cutting with T7 endonuclease I.

(b) Full - DNA gel showing the PCR product for each *crb2* F0 CRISPR mutant and control, before and after cutting with T7 endonuclease I.

Movie Titles and Legends

Movie 1: 3D-OCT imaging of the cranium (Stage 45 tadpole)

Movie 2: 3D-rendering of the ventricular system (Stage 45 tadpole)

Movie 3: Live imaging of the control - Stage 45 tadpole brain with OCT

Mid- sagittal view of the brain allowing simultaneous visualization of the four ventricles and intrinsic particles. Original acquisition (top) vertically stacked with the particle tracking movie (mid) and the stacked image (200frames) with temporal coding (bottom) to present particle trajectory over time. Based on the trajectory map, 5 distinct flow fields observed: FF1-FF5. We numbered flow fields 1 to 5 along to anterior- posterior axis. FF 1-Telencephalic ciliary flow and FF 2-Diencephalic ciliary flow are in the lateral and 3rd ventricle. FF 3-Mesencephalic ciliary flow belongs to the midbrain ventricle. Finally, the 4th ventricle has 2 flow fields, FF 4-Anterior Rhombencephalic ciliary flow and FF 5-Posterior Rhombencephalic ciliary flow (see also Figure 2) (30fps).

Movie 4: Live imaging of the *c21orf59* morphant

Mid-sagittal view of the brain allowing simultaneous visualization of the four ventricles and intrinsic particles. Original acquisition (top) vertically stacked with the particle tracking movie (mid) and the stacked image (200frames) with temporal coding (bottom) to present particle trajectory over time. On the first frame, cerebral aqueduct stenosis highlighted with a white arrow. Based on the particle tracking and the trajectory map, FF1-4 is absent (30fps).

Movie 5: Live imaging of the *foxj1* morphant

Mid-sagittal view of the brain allowing simultaneous visualization of the four ventricles and intrinsic particles. Original acquisition (top) vertically stacked with the particle tracking movie (mid) and the stacked image (200frames) with temporal coding (bottom) to present particle trajectory over time. Based on the particle tracking and the trajectory map, Stage 45 tadpole assessment shows the impairment of the flow fields in the lateral, 3rd and midbrain ventricle and slow flow in the fourth ventricle for the morphant as compared to the control (Movie 1) (30fps).

Movie 6: Live imaging of the *l1cam* F0 CRISPR mutant

Mid-sagittal view of the brain allowing simultaneous visualization of the four ventricles and intrinsic particles. Original acquisition (top) vertically stacked with the particle tracking movie (mid) and the stacked image (200frames) with temporal coding (bottom) to present particle trajectory over time. On the first frame, cerebral aqueduct stenosis highlighted with a white arrow. Based on the particle tracking and the trajectory map, 5 distinct flow fields remains present (30fps).

Movie 7: Live imaging of the *crb2* F0 CRISPR mutant

Mid-sagittal view of the brain allowing simultaneous visualization of the four ventricles and intrinsic particles. Original acquisition (top) vertically stacked with the particle tracking movie (mid) and the stacked image (200frames) with temporal coding (bottom) to present particle trajectory over time. Based on the particle tracking and the trajectory map, Stage 45 tadpole assessment shows the impairment of the flow fields in the lateral, 3rd ventricle and midbrain ventricle but normal flow in the fourth ventricle for the *crb2* F0 CRISPR mutant as compared to the control (Movie 1). White arrow on the first frame shows cerebral aqueduct stenosis as well as the absent flow fields (30fps).
